# Effects of mother-infant skin-to-skin contact on severe latch-on problems in older infants: a randomized trial

**DOI:** 10.1186/1746-4358-8-1

**Published:** 2013-03-11

**Authors:** Kristin E Svensson, Marianne I Velandia, Ann-Sofi T Matthiesen, Barbara L Welles-Nyström, Ann-Marie E Widström

**Affiliations:** 1 Department of Women‘s and Children's Health, Division of Reproductive Health, Karolinska Institutet, Stockholm, Sweden; 2 Karolinska University Hospital, Stockholm, Sweden; 3 Academy of Health, Care and Social Welfare, Mälardalen University, Västerås, Sweden; 4 Department of Educational Studies and Teacher Preparation, Graduate School of Education and Allied Professions, Fairfield University, Fairfield, CT, 06842, USA

**Keywords:** Breastfeeding emotions, Breast pain, Hands-on latch intervention, Latch-on problem, Infant, Innate breastfeeding program, Skin-to-skin

## Abstract

**Background:**

Infants with latch-on problems cause stress for parents and staff, often resulting in early termination of breastfeeding. Healthy newborns experiencing skin-to-skin contact at birth are pre-programmed to find the mother’s breast. This study investigates if skin-to-skin contact between mothers with older infants having severe latching on problems would resolve the problem.

**Methods:**

Mother-infant pairs with severe latch-on problems, that were not resolved during screening procedures at two maternity hospitals in Stockholm 1998–2004, were randomly assigned to skin-to-skin contact (experimental group) or not (control group) during breastfeeding. Breastfeeding counseling was given to both groups according to a standard model. Participants were unaware of their treatment group. Objectives were to compare treatment groups concerning the proportion of infants regularly latching on, the time from intervention to regular latching on and maternal emotions and pain before and during breastfeeding.

**Results:**

On hundred and three mother-infant pairs with severe latch-on problems 1–16 weeks postpartum were randomly assigned and analyzed. There was no significant difference between the groups in the proportion of infants starting regular latching-on (75% experimental group, vs. 86% control group). Experimental group infants, who latched on, had a significantly shorter median time from start of intervention to regular latching on than control infants, 2.0 weeks (Q_1_ = 1.0, Q_3_ = 3.7) vs. 4.7 weeks (Q_1_ = 2.0, Q_3_ = 8.0), (p-value = 0.020). However, more infants in the experimental group (94%), with a history of “strong reaction” during “hands-on latch intervention”, latched-on within 3 weeks compared to 33% in the control infants (Fisher Exact test p-value = 0.0001). Mothers in the experimental group (n = 53) had a more positive breastfeeding experience according to the Breastfeeding Emotional Scale during the intervention than mothers in the control group (n = 50) (p-value = 0.022).

**Conclusions:**

Skin-to-skin contact during breastfeeding seems to immediately enhance maternal positive feelings and shorten the time it takes to resolve severe latch-on problems in the infants who started to latch. An underlying mechanism may be that skin-to-skin contact with the mother during breastfeeding may calm infants with earlier strong reaction to “hands on latch intervention” and relieve the stress which may have blocked the infant’s inborn biological program to find the breast and latch on.

**Trial registration:**

Karolinska Clinical Trial Registration number CT20100055

## Background

Previous research shows that when a full term newborn is placed skin-to-skin on the mother immediately after birth, the newborn exhibits a pre-programmed biological behavior to approach the breast and start suckling without help [1,2]. During this first hour when the infant starts seeking the breast, the rooting reflex becomes successively more mature and distinct. During a mature rooting reflex, the mouth is wide open and ready to attach to the breast. At the same time, the tongue is positioned in the bottom of the mouth in order to be below the nipple/areola as the baby attaches to the breast with a forward movement with the head. Interestingly, prior to this rooting-tongue reflex [3], the baby makes licking movements, which are probably a program aimed at shaping the areola and nipple for easy attachment as well as to transmit taste from the breast to the baby’s mouth. This “familiarization” behavior can take up to 15 minutes before the baby attaches to the breast [4]. During this period, the baby usually massages the breast with the hands, which increases maternal oxytocin levels. This rise in maternal oxytocin is suggested to support milk ejection and maternal bonding [5].

Typical hospital staff practices that include trying to attach the infant to the breast with a grip around the infant’s neck and a grip around the mother’s breast/nipple (“hands-on latch intervention”) has earlier been suggested to cause an inhibition of the baby’s inborn rooting-tongue reflex [3]. Further, if this practice is too robust and intrusive the baby may scream and show an adverse behavior to the breast, fighting to avoid the breast instead of attaching to the breast to feed. Additionally, our clinical experiences over the years show that this kind of forceful help could be one underlying factor for infants’ latch-on problems. Further, Weimers et al. described mothers’ negative feelings about this practice. Many women have experienced the practice as unexpected when staff used hard-handed touching of the breasts when assisting the infant to attach to the breast [6]. This behavior can cause difficulties for the mother in understanding the infant and also undermine the mother’s self-confidence [7].

There are even many other labor and postnatal “routines” that are known to negatively affect breastfeeding and directly or indirectly cause breastfeeding problems. Some of these actions are: delayed first suckling [8,9] often caused by unnecessary separation of mother and infant [10]; supplementary feeds when not given for medical reasons [11,12] especially if the supplements are given by bottle instead of a cup to infants requiring multiple supplements [13]. Some other medical interventions like caesarean section, epidural and spinal anesthesia are often connected with delayed first suckling, breastfeeding problems and partial breastfeeding [14-17]. Thus infants with latch-on problems during the first months after birth are a common cause of parental and staff stress and may lead to early termination of breastfeeding [18-21].

Older infants with latching-on problems have not been studied previously with respect to the intervention of skin-to-skin contact with the mother. It is our hypothesis that when an older infant with latch-on problems is put skin-to-skin he/she will restore the pre-programmed biological behavior to attach to the breast and start sucking.

Thus, the overall aim of this study was to investigate if placing an older infant with severe latch-on problems skin-to-skin with the mother would positively affect the infants’ ability to latch-on when compared to those infants who did not have skin-to-skin contact but were held clothed in the mother’s arms in a common breastfeeding position.

The specific aims were to conduct a randomized study to compare experimental and control groups as to the following infant and maternal variables:

Infants: 1) Proportion latching-on; 2) Length of time until regular latching-on; and 3) Reaction to the hands-on latch intervention.

Mothers: 1) Assessment on Breastfeeding Emotional Scale; 2) Assessment on Breastfeeding Pain Scale; and 3) Experiences of “hands-on latch intervention”.

## Methods

The study was conducted between 1998–2004 at the Karolinska University Hospital in Solna, Sweden and Danderyds Hospital in Danderyd, Sweden. Both hospitals had about 5,000 deliveries per year. At these two hospitals healthy newborn infants were put skin-to-skin on their mother’s chest directly after birth and were not routinely exposed to oral or gastric suctioning. Two investigators, who were experienced practicing midwives as well as breastfeeding specialists, collected the data.

### Study design

**Figure 1:**
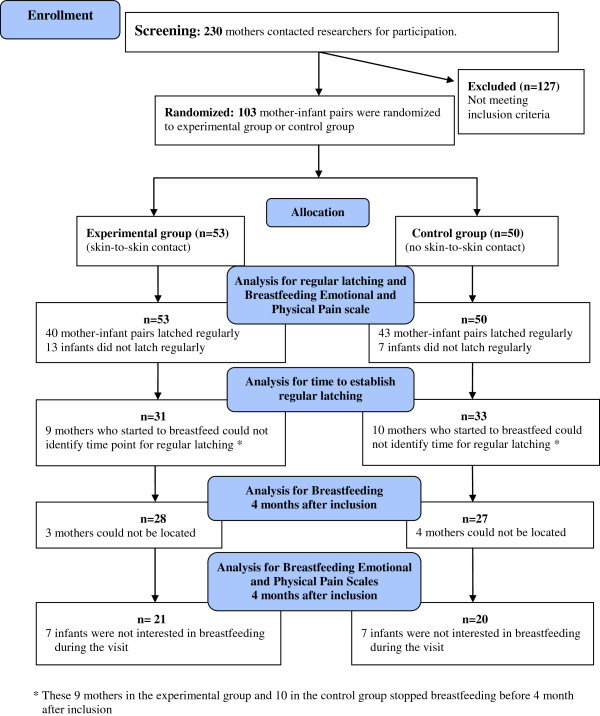
Flow diagram of progress through the phases of the study.

The study had a randomized block design and was performed at two sites, but was not conducted as a blind study. For an overview of the study design, see Figure 1.

### Research tools

For an overview of the administration of research tools see Table 1. To assess the mother’s experience of breastfeeding two tools were developed for the purpose of the study. The Breastfeeding Emotional Scale consisted of twelve 7-point semantic differential scales, the Breastfeeding Pain Scale of one. The words used as endpoints in the semantic differential scales were words that Swedish mothers often use to describe their breastfeeding experience. Four practicing breastfeeding counseling midwives validated the content of the samples of items. Fourteen mothers tested the scales and found them easily understood.

**Table 1:** Overview of methods and research procedure (with approximately required time in parentheses)

**First visit**	
Interview	Interview on background data and current breastfeeding situation (1 hour)
Self-rating scales	Breastfeeding Emotional Scale and Breastfeeding Pain Scale assessing feelings about breastfeeding in general (5 minutes)
Screening	Screening for latch-on problems during a breastfeeding session, including observation, assessment and consultation (30 minutes)
Randomization	Skin-to-skin during breastfeeding (experimental group) or in a common breastfeeding position during breastfeeding (control group)
Intervention	Breastfeeding session according to randomization (45 minutes) Breastfeeding Emotional Scale and Breastfeeding Pain Scale recalling feelings during the breastfeeding session (five minutes
**Visit after one week**	Support (varying time)
**Visit after four months**	
Interview	Questions about the current feeding situation (30 minutes)
Self-rating scales	Breastfeeding Emotional Scale and Breastfeeding Pain Scale (5 minutes)
Breastfeeding	Breastfeeding session (20 minutes)
Self-rating scales	Breastfeeding Emotional Scale and Breastfeeding Pain Scale (5 minutes)

#### Breastfeeding emotional scale

With the Breastfeeding Emotional Scale, mothers assessed their feelings on 12 subscales with the following end points representing opposite values; calm – stressed, unpleasant – pleasant, hopeful – hopelessness, fragile – strong, delighted – sad, meaningful – meaningless, fearful – not afraid, unconfident – confident, angry – harmonious, demanding – undemanding, manageable – unmanageable and contented – frustrated. Some statements had the positive word on the left side and others on the right side of the scale. Before the analyses were conducted, the positive word was converted and coded as a high number and then all numbers were summarized for each mother. High numbers were interpreted as a positive emotional response.

#### Breastfeeding pain scale

After mothers filled in the breastfeeding scale, they also assessed their feelings of pain related to breastfeeding before and during first breastfeeding attempt. The Breastfeeding Pain Scale was a 7-point semantic differential scale with words representing opposite values; severe pain - no pain. High numbers were interpreted as more pain. The scale was tested simultaneously with the Breastfeeding Emotional Scale.

#### Hospital records

Health and additional socio-economic background data including marital status and obstetric data, as well as data for infants, were collected from the antenatal and hospital records.

The Ethics Committee at the Karolinska University Hospital approved the study at both hospitals (Diary number 97–146). The trial was conducted before trial registration was standard practice, therefore it was registered after completion of the trial (Karolinska Triel Registry CT20100055).

### Research process

#### Inclusion/exclusion criteria

Healthy mothers who wanted to breastfeed and who had healthy infants with a severe latch-on problem (maternal assessment), which had not been resolved with previous medical consultation at either a hospital, well baby clinic or breastfeeding out-patient clinics in the hospitals’ catchment areas, were offered to participate in the study. Two infants with tongue-tie, which was released during the postnatal stay, were included in the study. A latch-on problem was considered when the infant 1) did not latch-on at all, or 2) had started to latch-on on but discontinued, or other serious problematic items identified by the women themselves had occurred as having infant who: 3) latched-on superficially without or with a nipple shield; or 4) latched only on one breast. For relevant terms and definitions see Table 2.

**Table 2:** Relevant terms and definitions

**Terms**	**Definitions**
Exclusive breastfeeding	The infant receives nothing but milk from the breast, vitamin D and essential medication
Hands-on latch intervention	Hospital staff, another person or relative uses their own hands to try to attach the infant to the breast with a grip around the infant’s neck and a grip around the mother’s breast/nipple sometimes using more or less force
Latch-on/latching	The infant is sustained attached to the breast with a wide open mouth over the nipple and the areola or parts of the areola and tongue in close contact with the lower part of the areola
Latching regularly	The mother perceives that the infant wants to breastfeed; the infant responds by latching-on the breast when it is offered
Nipple shield	Small plastic or rubber device shaped like the nipple that is placed over the nipple before latching
No separation	Mother and infant have skin-to-skin contact directly after birth and stay together during the first hour
Partial breastfeeding	Breastfeeding occurs regularly but the infant is also given supplementation or has started to eat solid food
Sucking	The infant is sucking the breast with rhythmic movements with pauses in between

#### Recruitment

Before and during the recruitment period, the staff at the postnatal wards at the two hospitals, well baby clinics and breastfeeding out-patient clinics, were informed about the study. A poster (size A4) information sheet was also put on the walls at the maternity wards and well baby clinics. The staff informed mothers who had infants with latch-on problems about the study, and the mothers themselves contacted the investigators about their willingness to participate at the hospital where their infants were born.

In total, 230 mother-infant dyads with latch-on problems contacted the investigators for participation in the study. An appointment was made for the mother-infant pair to meet the investigator collecting data at the hospital the infant was born. The meeting took place in a private room equipped with a bed, an arm chair and a table. Mothers were verbally informed by the investigators about the study and signed a consent form including assurances about ethical issues such as anonymity, and that they were free to discontinue the study at any time. During all procedures the comfort of the mothers and infants was a priority, so protocol for data collection took place only when the infant and mother felt at ease. All through the interview and breastfeeding session, the process was adapted to fit the mother’s and the infant’s individual situation. Thus, there was some flexibility in the order of the data collection but generally data was collected in the order described in Table 1.

#### Screening for severe latch-on problems

The two investigators (KES and MIV) developed a standardized assessment model in order to use a common clinical approach to guide mothers with breastfeeding problems. Reliability and consistency between the investigators and the information they gave was established. The model was applied during the interview and breastfeeding session and can briefly be described as follows: a) create a friendly and non-judgmental approach, b) be attentive and show an interest in listening, c) support the woman’s self-esteem d) discuss the infant’s signals, e) reinforce positive breastfeeding behaviors, and f) give knowledge-based information. Furthermore, the approach precluded the midwives touching either the infant directly, or the mother’s breasts. An outside reviewer found that there was reliability and consistency between the investigators and internal consistency regarding the consultation method they used to give mothers support, feedback and convey information about breastfeeding for both groups [22].

#### Interview

A semi-structured interview was conducted about the current breastfeeding situation, routines at the hospital and socio-economic and obstetrical background variables. This interview usually lasted between 1–1 ½ hour depending on the infants’ behavior, which could indicate that the mother and infant needed a break.

##### Breastfeeding emotional scale and breastfeeding pain scale assessing feelings about breastfeeding

To assess her feelings about breastfeeding, all mothers filled in the Breastfeeding Emotional Scale and Breastfeeding Pain Scale (see Research tools) before the screening breastfeeding session began.

##### Screening breastfeeding session, including observation, assessment and consultation

Mothers were asked to put the infants to the breast when they were ready. During the breastfeeding session, attention was paid to physical impediments to establishing good suckling behaviors, such as mother’s clothing or infant apparel or blankets. Then the mother’s body position and how she positioned the infant in relation to the breast, eye contact with the infant as well as the infant’s eye contact with the mother were noted. No infant was observed with tongue-tie. An observation protocol to identify infants with latch-on problems was developed. Each investigator tested the observation protocol on a select number of mothers simultaneously and independently. The protocol was revised and retested until reliability was established.

During the screening session, several types of infant latch-on on problems were observed and included such behaviors as no attempts to latch-on, failing to locate the breast, having the mouth wide open over the breast without attempting to latch-on, overactive rooting reflex over the breast, fending off the breast with their hands, or crying frenetically. Some infants looked like they wanted to avoid the breast, just fell asleep or did not move at all but remained in a “frozen” position.

##### Mothers and infants with resolved latch-on problems

In 127 mothers the latch-on problem was resolved during the screening process and those mothers went home. The collected data from these mothers and infants was not analyzed but stored for later analysis.

##### Mothers and infants with severe latch-on problems

During the screening 103 women were identified with a severe latch-on problem, which was not resolved at this first visit. Thus, these mothers were included in the study.

#### Randomization

The 103 mother-infant pairs with severe latch-on problems were randomly assigned to either an experimental or a control group as depicted in Figure 1. The randomization was performed at two hospitals and was blocked for time – eight consecutive mother-infant pairs in each block. Information about group inclusion was given in sealed opaque envelopes numbered from 1, 2, 3, 4, 5, 6, 7, 8, 9 . . . 60, which the investigator at each hospital opened consecutively in the mothers’ presence. At this time the mothers were given both oral and written information about details of their respective randomization group.

#### Intervention

After randomization, a breastfeeding session took place when the infant seemed to be ready to feed**.**

##### Experimental group

The skin-to-skin intervention was performed in the following way. The mother was in a hospital bed in a reclined position and she placed the infant prone between her breasts. The naked upper body of the mother was in contact with the infant’s naked body, although the infant could wear a diaper. If the mother felt cold, a blanket or sweater could be put on her shoulders to make her warmer or a little blanket could be put over the infant’s back. The mother was encouraged to allow the infant to crawl while holding a protective arm lightly over the baby and pillows supported her arms. The mother was encouraged to talk and communicate with her infant if she liked. If the infant began to cry the mother was encouraged to comfort the infant as she saw fit, even if it meant sitting up to comfort her infant. Once the infant was calm again, she was encouraged to return to skin-to-skin contact in a reclined position.

In addition, mothers in the experimental group were encouraged to use skin-to-skin as often as they wanted when practicing breastfeeding at home. They were asked to document each time skin-to-skin was practiced.

##### Control group

In the control group, mothers and infants were fully dressed. The mother was usually positioned in the common breastfeeding position sitting in an armchair holding the infant in front of the exposed breast. She was advised to start breastfeeding as she usually did. If the mother felt more comfortable lying down she could do so. The mother was encouraged to allow the infant to move his/her head and arms freely. The mother’s arms were supported with pillows. The mothers were encouraged to talk and communicate with her infant if she liked. If the infant began to cry the mother was encouraged to comfort the infant as she saw fit. Once the infant was calm again, she was encouraged to try breastfeeding again.

##### Both groups

Immediately after the intervention, the mothers in both groups filled in the same two scales on her breastfeeding emotions and pain related to breastfeeding, recalling her feelings during this breastfeeding session. All mothers were instructed to apply the knowledge they had gained during this breastfeeding session when they returned home. Mothers were given a new appointment with the investigator in the same setting, at one week and four months after inclusion.

#### Follow-up visits

At one week, mothers were asked relevant questions about her current feeding situation. The investigators also offered appropriate individual support if needed.

At four months, mothers still breastfeeding filled in the Breastfeeding Emotional Scale and the Breastfeeding Pain Scale again to evaluate their feelings before and during a breastfeeding session at this visit.

### Statistical analysis

Nominal data are presented as proportions. Ordinal data are presented by median and quartiles (Q_1_, Q_3_). Interval data are presented by mean and standard error. For nominal data, differences between treatment groups were tested by Fisher’s exact test. The Mann–Whitney U-test was used comparing groups both for ordinal and interval data. Data based on added items (Breastfeeding Emotional Scale) were tested by t-tests.

Simple regression analyses were used to study the relationship between the dependent variable *time when regular latching and suckling occurred* and the two independent variables *infant’s age at* and *the number of skin-to-skin events* (only for treatment group, data not shown). A p-value ≤ 0.05 was considered significant.

Survival analysis was done using the Kaplan-Meier method (the Peto-Peto-Wilcoxon test was used to compare the curves).

Due to the time-consuming screening procedure during which an unforeseen large number of mothers and infants were cured from the latch-on problem, we had to stop data collection earlier than planned. Originally we planned to have 60 observations in each treatment group but had to use 50 instead.

The calculations of power are based on testing the hypothesis that the true difference between the two proportions p_1_- p_2_ are 0 against the alternative hypotheses that this difference is greater than 0.20. When computing variances for proportions we used the fact that the maximum variance for a proportion occurs when the proportion equals 0.5. For n = 50 in each group the maximum variance of p_1_-p_2_ is 2(0.5(1–0.5)/50) = 0.01.

Using this as an upper limit for the variance both for H_0_ and H_1_ the calculated power of the test will be more than 84% when using 50 observations in each group.

## Results

### Background data

There were no significant differences between the two groups in terms of background variables (Table 3), delivery and postnatal ward experiences (Table 4), mother’s experience and description of the infant’s reaction to “hands-on latch intervention” (Tables 5 and 6), infant age at trial entry, nutrition and way of giving breast milk /supplements, use of pacifier, nipple shield and maternal feeding intentions (Table 7).

**Table 3:** Socio-economic and obstetric background data collected from medical records

**Characteristics**	**Experimental** **group n = 53** **No. (%)**	**Control group** **n = 50** **No. (%)**
**Mothers**		
Age (years) Md (Q_1, 3_)	31 (29–34)	33 (29–35)
Primipara	43 (84)	36 (72)
**Education**		
Primary	4 (8)	4 (9)
Secondary	18 (37)	17 (36)
University	27 (55)	26 (55)
**Civil status**		
Married or cohabiting	50 (98)	48 (98)
**Obstetrical data**		
Pre-eclampsia	8 (16)	7 (15)
Induction of labor	11 (22)	6 (12)
Normal vaginal delivery	23 (45)	31 (62)
Vacuum extraction	7 (14)	3 (6)
Forceps	1 (2)	0 (0)
Caesarean section birth		
Elective	9 (18)	10 (20)
Emergency	11 (22)	6 (12)
Presentation		
Head	44 (88)	44 (90)
Breech/foot	6 (12)	5 (10)
Oxytocin infusion	39 (83)	29 (73)
Meconium stained in amniotic fluid	12 (24)	15 (31)
Ruptured membranes >24h	6 (12)	7 (15)
Anesthesia^*^		
No anesthesia	12 (12)	14 (14)
General anesthesia	2 (4)	2 (4)
Epidural block	21 (41)	16 (31)
Spinal block	16 (31)	13 (27)
Pudendal block	4 (8)	2 (4)
Nitros oxide	25 (51)	33 (70)
Bleeding postpartum (ml) Md (Q_1, 3_)	465 (317–700)	405 (310–582)
**Infant**		
Gestational age (weeks) Md (range)	39 (34–42)	39 (34–41)
Birth weight (g) Md (range)	3460 (2130–4790)	3500 (2070–4490)
Gender female	29 (55)	27 (54)
Healthy full-term, Apgar >7 at 5 min	38 (78)	40 (85)
Full-term, Apgar ≤7 at 5 min	4 (8)	0 (0)
Healthy premature ≤ week 37 Apgar >7 at 5 min	7 (14)	6 (13)
Premature ≤ week 37 Apgar <7 at 5 min	0 (0)	1 (2)
Infant cared for in the neonatal ward	10 (20)	3 (6)

^*^ Some mothers had more than one types of anesthesia.

**Table 4:** Hospital data for mothers and infants

	**Experimental** **group** **n = 53** **No. (%)**	**Control group** **n = 50** **No. (%)**
**Care practices**		
Oxygen postpartum	14 (28)	10 (20)
Oral or gastric suction postpartum	19 (39)	16 (34)
No separation first hour postpartum	28 (55)	28 (58)
Breastfeeding attempt ≤2 hour postpartum	16 (31)	19 (40)
**Infant**		
**Complications first days postpartum**		
Hypoglycemia	14 (27)	14 (29)
Jaundice	11 (22)	12 (24)
Weight loss ≥10%	8 (16)	8 (16)
Blocked/obstructed nose/ breathing	2 (4)	3 (6)
Swollen tongue	1 (2)	0 (0)
Occasional vomiting first days	19 (44)	13 (31)
Tongue-tie	0 (0)	2 (4)
**Mothers**		
**Noted “physical problems” from the record**		
Breast/nipple status		
-sores or/and severe nipple pain	7 (15)	7 (15)
-inverted, flat nipple	16 (32)	24 (51)
-previous breast reduction	3 (6)	3 (6)
**“Hands-on latch intervention” during hospital stay**		
-yes	43 (93)	44 (96)
**Supplementary feeding**		
Formula	42 (84)	39 (78)
Supplement days Md (Q_1, 3_)	2 (0–4)	2 (0–3)
Reason for supplement feeding		
Medical reason	29 (58)	24 (48)
No reason given	13 (26)	15 (30)
**Maternity stay,** (hour) Md (Q_1, 3_)	120 (102–141)	114 (84–135)

^*^ Some infants had more than one complication.

**Table 5:** Examples of “mothers' reports of 'hands-on latch intervention' during hospital stay”

**Mothers reports of “hands-on”**	**Experimental** **group** **n = 53** **Number of** **mothers**	**Control group** **n = 50** **Number of** **mothers**
**“Hands-on” not hard:** “not painful” “I didn’t know anything else” “unpleasant” “stressful”	19	19
**“Hands-on” hard:** “squeeze and pulling hard” “painful” ”violent” “humiliating” ”forceful” “heavy handed” “lasting bruises”	24	25
**Did not receive “hands-on”**	3	2
**Not noted**	7	4
**Total**	53	50

**Table 6:** Examples of "mothers' descriptions of infants' reactions during hospital stay"

**Mothers descriptions of infants reaction on “hands-on”**	**Experimental** **group** **n = 53** **Number of** **infants**	**Control group** **n = 50** **Number of** **infants**
**Suckled temporarily**	0	1
**Passive reaction**	11	9
“no reaction” “passive” “fell asleep” “didn’t care” “not interested” “bored” “turned off”		
**Strong reaction**	30	32
“screaming” “became hysterical” “was defensive” “avoidant” “sad” “panicked” “worried” “mad” “angry”		
**Did not receive hands-on intervention**	3	2
**Not noted**	9	6
**Total**	53	50

**Table 7:** Data on mothers and infants at the time of entering the study

	**Experimental** **group n = 53** **No. (%)**	**Control group** **n = 50** **No. (%)**
**Infants**		
Age of the child (weeks) Md (Q_1, 3_)	3.0 (2.0-6.2)	2.7 (1.7-4.3)
**Latching and suckling ability according to the mothers**		
No latching or suckling at all	34 (64)	29 (58)
Superficially latching	2 (4)	4 (8)
Superficially with nipple shield	15 (28)	13 (26)
Latching only on one breast	2 (4)	4 (8)
**Nutrition**		
Breastfeeding with nipple shield	2 (4)	4 (8)
Only breast milk (expressed)	16 (30)	14 (28)
**Supplement/breast milk given by:**		
Bottle	42 (79)	34 (68)
Alternative methods:		
-cup	7 (13)	10 (20)
-similar to cup	1 (2)	2 (4)
**Use of pacifier**	18 (40)	12 (32)
**Mothers**		
**Mothers intended to breastfeed**		
less than 6 months	3 (8)	4 (10)
6 months	10 (26)	17 (41)
more than 6 months	25 (66)	20 (49)

#### Breastfeeding emotional scale

There were no significant differences between the experimental and control groups in mothers’ mean score on the Breastfeeding Emotional Scale administered before the screening breastfeed session (mean 58.3, SE 1.85 vs. mean 56.2 SE 2.23, p-value = 0.470).

#### Breastfeeding pain scale

There were no significant differences between the experimental and control group on the mothers’ score on the Breastfeeding Pain Scale administered before the screening, breastfeed session median 1.0 (Q_1_ = 1.0 – Q_3_ = 4.8) vs. median 1.0 (Q_1_ = 1.0 – Q_3_ = 4.0) p-value = 0.540.

### Data after intervention

#### Breastfeeding emotional scale

The mean scores on the Breastfeeding Emotional Scale assessed during the breastfeeding session after intervention was significantly higher in the experimental group than in the control group (mean 69.2, SE 1.68 vs. mean 62.4, SE 2.44, p-value = 0.022).

#### Breastfeeding pain scale

During the breastfeeding session after intervention, the mothers in the experimental group scored significantly lower on the Breastfeeding Pain Scale than the control group, median 1.0 (Q_1_ =1.0 – Q_3_ = 1.0) vs. median 1.0 (Q_1_ = 1.0 – Q_3_ = 2.0) p-value = 0.044.

#### Proportions of infants starting regular latching-on and suckling

The proportion of infants starting to latch-on and suckle did not differ significantly between the experimental group 75% (n = 40), and control group 86% (n = 43) (Fisher’s Exact test p-value = 0.217).

#### Time from intervention to regular latching and suckling

**Figure 2:**
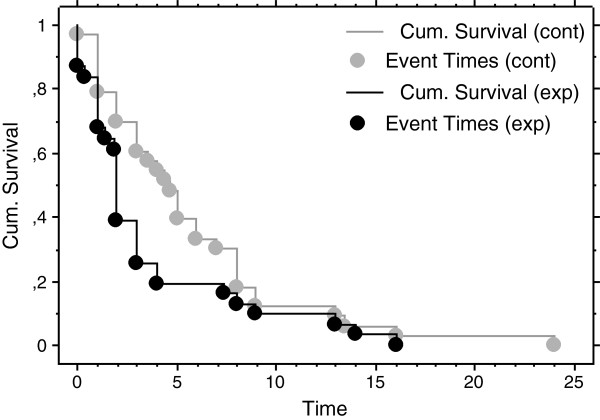
Kaplan-Meier curves for comparing cumulative survival and event in weeks from intervention to infants regular sucking and latching in the control group (gray lines and circles) and in the experimental group (black lines and circles).

For different reasons, 9 mothers in the experimental group and 10 in the control group could not tell the time lapse between intervention and regular latching. (See Figure 1 and Discussion of methods). The 31 infants in the experimental group with registered time began latching on in a significantly shorter median time than the 33 infants with registered time in the control group, 2.0 weeks (Q_1_ = 1.0, Q_3_ = 3.7) vs. 4.7 weeks (Q_1_ = 2.0, Q_3_ = 8.0), (p-value = 0.020). To illustrate the differences between the groups a Kaplan-Meier time to event curve was made (p-value = 0.017, Peto-Peto-Wilcoxon test) (Figure 2). In addition, it was found that 23 infants out of the 31 (74%) in the experimental group started to latch regularly and suckle within three weeks versus 13 infants out of the 33 (39%) in the control group (Fisher’s Exact test p-value = 0.006)**.** When trying to find a possible reason for this difference we explored if there may be a relation to infants’ reaction to “hands-on latch intervention”. It was found that in the experimental group 94% of those infants starting to latch on regularly and suckle within three weeks had a history of “strong reaction” during “hands-on latch intervention” compared to 33% of the infants with “strong reaction” in the control group who started to latch on within 3 weeks (Fisher’s Exact test p-value = 0.0001). A similar number of infants in the experimental and control group had shown a “strong reaction” to the “hands-on latch intervention” (Table 6).

#### Infants age in relation to time for regular latching and suckling

**Figure 3:**
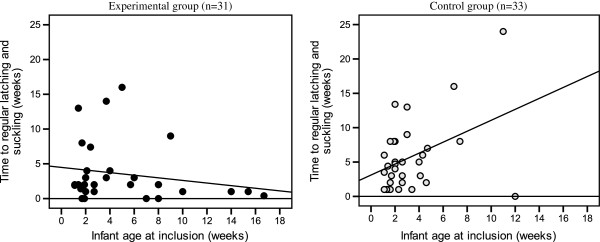
Descriptive regression plots for experimental and control groups showing infants age in weeks at inclusion and time (weeks) it took to established regular latching and suckling.

To illustrate if age of the infants at inclusion was related to the time it took to establish regular latching and suckling two simple regression analyses were performed. It was found that in the control group the infant age at inclusion correlated positively and significantly with the time it took to establish regular suckling (R = 0.409, p = 0.018, R^2^ = 0.167). Thus, the older the infant, the longer the time to regular latching. In the experimental group there was no significant correlation (R = 0.192, p-value = 0.300, R^2^ = 0.037) (Figure 3).

#### Number of skin-to-skin contacts in relation to time for regular latching and suckling

The median number of skin-to-skin contacts between mother and infant in the experimental group during the first 7 days after the intervention was 8.5 (Q_1_ = 3.0, Q_3_ = 14.5) times. To understand if the number of skin-to-skin contacts affected the time it took to establish regular latching and suckling a simple regression analysis was performed. It was found that the number of skin-to-skin contacts did not correlate significantly with time elapsing from intervention to the time infants started to regularly latching and suckling (R = 0.158, R^2^ = 0.025, p-value = 0.472).

#### Mother’s response to infant’s regular latching and suckling

Unexpectedly, 4 mothers in the experimental group and 8 in the control group stopped breastfeeding when the infants started regular latching and suckling. Nor did these mothers give their infants expressed breast milk after they discontinued breastfeeding.

Sixty-three percent (25/40) of the infants in the experimental group who started to latch-on and suckle were also exclusively breastfeed compared to 58% (25/43) of the mothers in the control group (p-value = 0.685). Among mothers whose infants did not latch-on at all, 11 in the experimental group and 4 in the control group continued to express breast milk and feed the infant by bottle.

### Four months follow up after inclusion

#### Breastfeeding

Of those mothers whose infants started latching-on and suckling regularly 73% (n = 29) in the experimental group and 63% (n = 27) in the control group were continuing to breastfeed four months after intervention (p-value = 0.284).

#### The median age of the infants when starting latching-on and suckling regularly

The median age of the infants when starting to latch-on and suckle regularly was 7.7 weeks, (Q_1_ = 3.7, Q_3_ = 10.7) in the experimental group vs. 7.1 weeks, (Q_1_ = 4.6, Q_3_ = 11.7) in the control group. This difference was not significant (p-value = 0.919).

#### Breastfeeding emotional scale

At four months after inclusion, mothers who were still breastfeeding filled in the Breastfeeding Emotional Scale once more before and during breastfeeding. For different reasons, seven of the breastfeeding mothers in each group did not have the opportunity to fill in the scale (Figure 1 and Discussion of methods).

There was no significant difference in the mean scale scores between the experimental group, (n = 21) and the control group (n = 20) either before (72.6, SE 2.05 vs. 74.7, SE 2.73) nor during breastfeeding (72.2, SE, 2.65 vs. 75.3, SE 2.60) four months after inclusion.

## Discussion

The main finding of this study was that among those infants who started regular latching and suckling it was found that the infants randomized to receive skin-to-skin contact with the mother during breastfeeding (experimental group) spent a significantly shorter median time lapse between start of treatment and commencement of regular latching and suckling compared to the group of infants who were not put skin-to-skin with their mothers during breastfeeding (control group). Both groups of mothers had received breastfeeding counseling according to a standardized assessment model (see Screening for severe latch-on problems). Thus, skin-to-skin contact seems to have added a positive effect on the infants potential to latch-on and may be specifically beneficial to infants with a history of having reacted strongly (panicked, frenetic crying) when “helped” by staff to be attached to the breast with “hands-on latch intervention.”

In addition, skin-to-skin contact initiated positive maternal feelings during the breastfeeding session with skin-to-skin, after the intervention. The same immediate rise in maternal feeling could not be seen in mothers in the control group during the attempt to breastfeed after the intervention.

Unexpectedly, some mothers in both groups stopped breastfeeding after the infants had started to latch-on.

### Discussions of methods

All mothers in this study had been seeking help for the infants’ difficulty to latch-on to the breast at postnatal ward, well baby clinics and breastfeeding out-patient clinics. Within one screening visit, the two investigators in this study solved the sucking problems in 127 of the 230 mother- infant pairs. This implies that the method used to guide mothers was well adapted to the mother’s breastfeeding problem. The same method for breastfeeding counseling was used in both the experimental and control group. Approximately the same number of infants started to suckle in both the experimental and the control groups, even when controlling for hospital/midwife. Thus, in addition to this method for counseling, skin-to-skin contact between mother and infant seems to have added some important factors, as the infants in the skin-to-skin group started to suckle regularly after a significantly shorter median time than the infants in the control group.

#### Considerations on sample size

The issues of longitudinal studies on breastfeeding include the diminishing number of participants over time. Of the 103 mother-infant dyads taken in to the study, 83 infants (81%) started to latch on and suckle regularly.

#### Time to regular latching and suckling

Among those 83 mothers with infants who started regular latching and suckling, 9 mothers in the experimental group and 10 mothers in the control group could not specify the exact time point when the infant started regular latching and suckling. When comparing background data (maternal age, civil status, education, caesarean birth, spinal or epidural anesthesia, birth weight, sex and parity), there were no significant differences between those mothers who could and could not specify the exact time point.

Interestingly, there might be reasons for why these mothers could not specify the exact time point. Six of them (3 in each group) continued using nipple shields and that might have made it more difficult to specify the exact time point. The remaining mothers (6 experimental mothers and 7 control mothers) who could not specify the exact time point for latching and suckling went on with giving formula by bottle. This may have made it difficult for them to distinguish between irregular and regular latching and suckling. However, the number of mothers and the tentative explanations for why they could not specify the time for regular latching is evenly distributed in experimental and control group.

#### Unexpected cessation of breastfeeding

Seven partly breastfeeding mothers stopped breastfeeding just when the infant started to latch-on and suckle regularly. The reason for this is not known, but hypothetically they may have belonged to a group of mothers who intellectually knew the advantages of breastfeeding but may have had difficulties with touching. As described by Wood and Van Esterik, sometimes the phenomenon of touching, be it self touch, the infant’s touch, or medical touching of the breast, can negatively affect women who then cannot overcome these negative feelings when intending to breastfeed [23].

#### Interviews and scales

The main reason for internal missing data in Interview questions and Breastfeeding Emotional Scale and Pain Scale (see Tables 5 and 6 and Figure 1) was that in some situations, especially when the baby was fussy and difficult to comfort, it felt unethical to force the mother to complete interviews and questionnaires. In addition, some of the infants continuing to breastfeed were not interested in sucking the breast during the follow-up visit four months after entering the study. Thus, in those cases the Breastfeeding Emotional Scale and Breastfeeding Pain Scale could not be filled out. However, there were no significant differences in background variables: maternal age, civil status, education, caesarean birth, spinal or epidural anesthesia, birth weight, sex and parity between the mothers who answered or did not answer the questionnaires Breastfeeding Emotional Scale, Breastfeeding Pain Scale, the interview about “hands-on latch intervention” (Table 5) and the infant’s reaction on “hands-on latch intervention” (Table 6). There was no significant difference between the experimental or control group in number of mothers not filling in scales or giving answers to questions.

### Discussion of the results

Important to note in this study are that the mother-infant dyads had above average rates of epidural, caesarian, separation after birth, supplementation, “hands-on latch intervention” and the infants were bottle fed when they entered the study. In spite of mother-infant experience during hospital stay and infant’s age, the majority of the infants started latching-on and suckling regularly.

#### Infants’ reaction to skin-to-skin contact

The reason that the infants in the skin-to-skin group started to latch on in a significantly shorter median time than the group of infants dressed in the mothers’ arm may be that they re-capitulate the inborn biological behavior to find the mother’s breast, got acquainted with the breast by licking and touching in their own way when being able to move freely on the mother’s chest. It is suggested that during this process the infant develops the rooting-tongue reflex. During this reflex the infant opened up the mouth widely and put its tongue in the bottom of the mouth [3] in order to be able to latch-on to the breast and start suckling. This assertion is supported by a study where it was shown that significantly more infants among those who had uninterrupted skin-to-skin contact for at least one hour after birth were sucking correctly compared to infants who had been separated after 20 minutes and then returned clothed to the maternal breast after finishing with hospital care routines, such as being weighed, washed, etc. [24].

Infants in the experimental group in the present study who had “reacted strongly”, were described by the mothers as “screaming hysterically” during “hands-on latch intervention”, were those who started to suckle significantly earlier than the infants in the control group who had “reacted strongly” to the “hands-on latch intervention”. Our interpretation is that the skin-to-skin contact with the mother induced calmness in the infant. Bystrova et al. suggested that skin-to-skin early after birth decreases the negative effects of “the stress of being born” [25]. It is likely that skin-to-skin contact with the mother even in these older infants will counteract “the stress of being forced to breastfeed”. This is supported by a recent study that infants staying skin-to-skin for more than one hour after birth had lower cortisol levels in saliva than infant staying less than one hour, indicating that skin-to-skin contact relieves stress and thus induces relaxation in the infant [26]. Skin-to-skin contact may relieve the memory of stress and possible experienced “suffocation” in those infants who were forced to the breast by a heavy-handed person. With skin-to-skin contact, the infant can relax, stop crying and maintain enough calm to successively, and successfully, co-ordinate body movements with the five senses (sight, touch, hearing, smell and taste) to achieve the latch and suckle, echoing the patterns of a normal newborn during the first hours of life [4]. The infants in the control group where mothers held their infant in a common breastfeeding position and the infant was dressed may have had limited possibility in this respect.

Interestingly, it has previously been found that infants who remained skin-to-skin with their mother one and a half hours after birth had more optimal self-regulation/organizational capacities [27] one year later than infants who were separated from their mothers and placed in the nursery during the same length of time [28]. It is possible that the skin-to-skin sessions with the mother even helped the older infants in the present study to a more optimal self-regulation, which may have helped the infant to control the breast-feeding situation. Infants who were clothed and placed in front of the breast in a common breastfeeding position were possibly more restricted in movements when trying to attach to the breast, like the infants in the control group. Presumably neither the infant nor the mother could fully relax in this group. The infant’s stress may have affected the mother; and mother’s the stress may also have affected the infant.

#### Mothers’ reaction to skin-to-skin contact

Skin-to-skin contact may have relieved maternal stress as we anecdotally have observed in this study, as well as in clinical practice, that some mothers’ milk starts to leak when the infants are put skin-to-skin. The skin-to-skin contact may cause an increase in oxytocin release followed by relaxation and subsequent milk letdown [29]. The fragrance of the milk may have stimulated the infant to seek the breast. To see the milk “flowing” and the infant’s attraction to the breast may have another positive effect on the mother; it may have stimulated her sense of self-confidence.

Oxytocin is also looked upon to be “the love hormone” and to elicit feelings of relaxation and calmness in breastfeeding mothers [30]. In line with this, is that mothers in the skin-to-skin group rated significantly higher on the Breastfeeding Emotional Scale already during the first breastfeeding in skin-to-skin contact with the infant, than did the control mothers who breastfed in the common position. Thus the skin-to-skin contact with the infant seems immediately to have enhanced maternal positive emotions towards breastfeeding. This suggests that the mothers, as well as the infants, in the experimental group were relaxed by skin-to-skin contact.

In addition, the mothers in the skin-to-skin group described significantly less pain during the first breastfeeding in skin-to-skin contact. This result may have to do with the pain-relieving effect of skin-to-skin contact. Similar effects of skin-to-skin contact have been seen in newborns in a previous study [31]. A relaxed mother with low pain may open up mentally during breastfeeding and be able to better interpret and understand the signals of the infant [32,33].

Interestingly, after four months, mothers who continued to breastfeed in the control group had reached about the same level of positive feelings on the Breastfeeding Emotional Scale as those continuing breastfeeding in the experimental group indicated already after the first skin-to-skin session.

#### Process of initiating latch-on

These described mechanisms may explain why the process of initiating successful latch-on is quicker in the group of infants in the skin-to-skin group than in the control group. A large number of infants in this study with diagnosed latch-on problems had a history of having been attached to the mother’s breast by the health workers with “hands-on latch intervention” and showed, according the mothers, a “strong negative reaction” of screaming. Placing infants skin-to-skin with the mother immediately after birth may preclude the need for heavy-handed latch-on intervention because the infant will develop the biological breastfeeding reflexes by him/herself [3] leading to an optimal latch [29]. Thus, to minimize latch-on problems in the future, healthy newborn infants merely need to remain skin-to-skin with the mother immediately after birth, and periodically during the postnatal stay, in order to be able to start breastfeeding when breastfeeding reflexes are well developed.

## Conclusions

Similar number of mother-infant dyads in both groups started to latch on and suckle regularly. On the other hand, mothers in the skin-to-skin group were relieved from the stress of having a breastfeeding problem twice as quickly as mothers in the control group.

Infants who experienced skin-to-skin and had a history of “strong reaction” to “hands-on latch intervention” were those restoring the biological breastfeeding program most rapidly. The stress of being forced to the breast was possibly a contributing factor to the infant’s latching-on problem. The skin-to-skin contact may have decreased the stress and induced a therapeutic relaxation in both mother and infant during which the infant was able to restore the biological program of breastfeeding behavior. Furthermore, mothers in the skin-to-skin group rated more positively on the Breastfeeding Emotional Scale during the breastfeeding session after intervention with skin-to-skin, than did the control mothers.

Clinical implications of our findings suggest that mothers who want to breastfeed and have an older infant with a latch-on problem should be supported not to give up breastfeeding since the age of the infants in this study who began breastfeeding ranged from two weeks up to around five months. Since the problem is resolved more quickly during skin-to-skin contact, the results support that skin-to-skin intervention should be considered a choice of treatment to rectify long-lasting latch-on problems. Latch-on problems may possibly be prevented if infants and mothers have skin-to-skin contact directly after birth until the infant has developed the biological breastfeeding program and started suckling without help.

The issues of longitudinal studies on breastfeeding include the diminishing number of participants over time. This was also the case in this research study. Therefore we suggest that these data should be interpreted with caution. We suggest that further studies of the origins of latch-on problems be conducted.

## Competing interests

The authors declare that they have no competing interests.

## Authors' contributions

The study was and designed in collaboration with KES, MIV, AMEW, and ASTM, MIV, AMEW and KES constructed and pilot-tested the self-rating scales and research protocols. KES and MIV collected all data. KES shared the main responsibility for statistical analyses with ASTM, AMEW and MIV. KES wrote the manuscript with AMEW, MIV, BLWN and ASTM. All authors read and approved the final manuscript.
